# Roles of Mature Domain Targeting Signals (MTSs) for Protein Translocation and Secretion in *Lactococcus lactis*

**DOI:** 10.3390/ijms26010219

**Published:** 2024-12-30

**Authors:** Mai Ngoc Hoang, Clemens Peterbauer

**Affiliations:** Institute of Food Technology, Department of Food Science and Technology, BOKU University, 1190 Vienna, Austria

**Keywords:** *Lactococcus lactis*, secretory proteins, signal peptide, mature domains, hydrophobic patches, SecA

## Abstract

*Lactococcus lactis* is a potential bacterial cell factory to develop delivery systems for vaccines and therapeutic proteins. Much progress has been made in applications using engineered *L. lactis* against, e.g., inflammatory bowel disease and cervical cancer, but the improvement of secretion and cell anchoring efficacy is still desirable. A double-labeling method based on biarsenical hairpin binding and nickel–polyhistidine affinity was used for visualization of protein trafficking and the quantification of targeted proteins on the cell surface and in the cytoplasm. To investigate the importance of mature domain targeting signals (MTSs), we generated truncated constructs encoding 126, 66, and 26 amino acid residues from the N-terminus of the basic membrane protein A (BmpA) and fused those with the gene for the human papillomavirus serotype 16 (HPV16) E7 oncoprotein. Overexpression of fusion proteins was observed to come at the cost of cell proliferation. *L. lactis* cells produced and displayed the shortest fusion protein only with difficulty, suggesting that the entire absence of a homologous sequence containing MTSs significantly impedes the export and surface anchoring of fusion proteins. With 40 amino acids following the signal peptide and containing one MTS, effective translocation was possible. Mutations of MTSs towards increased hydrophobicity resulted in increased secreted and surface-displayed fusion protein, suggesting the potential to design rationally improved constructs.

## 1. Introduction

*Lactococcus lactis,* a naturally safe microorganism for daily consumption in food, has been emerging as a potential medical cell factory and drug delivery vehicle. Among the advantages of the utilization of this bacterium in such applications are its simple energy metabolism, modest genome scale, various available expression systems, and limited complications (e.g., no lipopolysaccharide, only one housekeeping protease, etc.). Up until now, at least 100 recombinant proteins from various hosts have been efficiently produced in *L. lactis* merely using the constitutive P170 expression system [1]. Mammalian-derived proteins were produced successfully, including bovine plasmin [2], bovine β-Lactoglobulin [3], murine interleukin-2, and interleukin-6 [4,5], etc. Heterologous proteins from other prokaryotic species (e.g., *Staphylococcus hyicus* or *Brucella abortus*) comprise enzymes such as lipase [6], bacteriophage lysins [7], or immunogenicity factors such as *Brucella abortus* antigen L7/L12 [8]. The yield of these products was also optimized impressively; for instance, nuclease from *S. aureus* was overexpressed in *L. lactis* at yields of 2.5 g/L in batch fermentation [1].

The targeting of secretory proteins to translocons was believed for many years to be uniquely performed by signal peptides [9,10], but it was later revealed to also be influenced by targeting signals concealed in mature domains of preproteins [11,12,13]. These investigations explain the findings that in some cases, signal peptides are not able to facilitate the secretion of heterologous proteins fused to them [13] and that some preproteins can be secreted in the absence of a signal peptide [12,14,15]. Important roles of the mature domains of secretory proteins were speculated to involve SecA lipid stimulation, SecA translocation [16], and ATPase activation [15,16] by a possible specific binding affinity with SecA [15]. Mature domain targeting signals (MTSs) is a term first used by Chatzi et al. for these signal-peptide-independent targeting signals [11]. In *E. coli*, it was shown in vitro that these domains were able to function either independently [11,12,17] or complementarily with signal peptides for the trafficking of preproteins to membrane-bound translocons [15,17]. MTSs are identified as multiple hydrophobic patches of each protein sequence. The individual presence of a single MTS, co-existence of several MTSs, and the combination with or without native signal peptides significantly affects binding affinity of preproteins with SecA-SecYEG in vitro [11]. The aim of this study is to examine the influence of MTSs in protein secretion efficiency in *L. lactis* as the model organism for lactic acid bacteria. Here, we introduced genetic modifications to physically alter the hydropathy profiles of several MTSs in silico and utilized in vivo techniques to evaluate secretion efficiency in *L. lactis*. In-depth knowledge about the properties and role of MTS in several cellular pathways may pave the way for the rational design of heterologous proteins and fusion proteins for improved secretory production in bacterial cell factories.

## 2. Results

### 2.1. Effects of Fusion Protein Overproduction on Cell Growth

**Figure 1:**
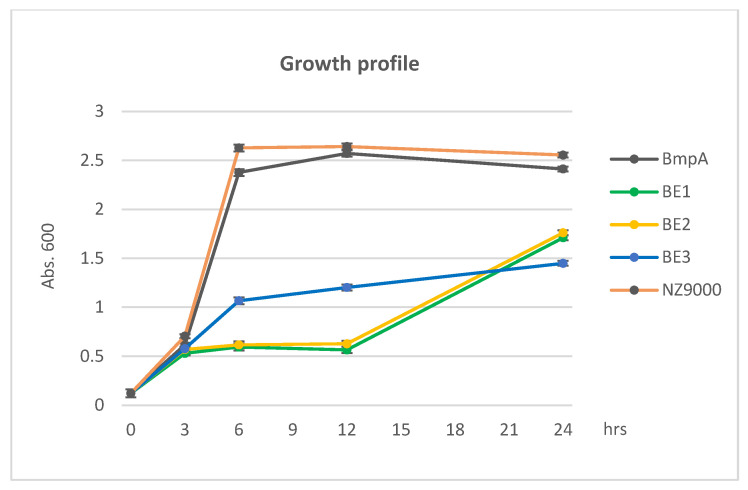
Growth profile of *L. lactis* carrying homologous proteins and fusion proteins. Optical density (OD) at 600 nm wavelength was measured after 3-, 9- and 24-h post-induction.

*L. lactis* BmpA is classified as a lipoprotein that is covalently attached to membrane lipids via a conserved cysteine after cleavage of the signal peptide [18]. The predicted cleavage site is between position 21 and 22; the conserved Cys residue is at position 22. We chose 26 amino acids for the shortest construct (BE3), comprising only the SP, plus five amino acids including the Cys residue, to facilitate membrane attachment, followed by the heterologous fusion partner. Construct BE2 comprises an additional 40 amino acids, and BE1 comprises an additional 100 residues, in order to assess the effect of increased lengths of the mature domain of the fusion partner on secretion and display efficiency, as well as on potential negative side effects. Growth of all the cell lines was similar in the pre-induction period, but the growth of cells producing full-length homologous proteins surpassed that of cells carrying fusion constructs with the heterologous protein after the inducer nisin was introduced. Until three hours post-induction, exponential growth was observed in BmpA-producing cells, similar to the native NZ9000, which also reached the cell density peak between three and six hours. A slower increase was seen in cells carrying the shortest homologous domain, BE3. Very slow growth was observed in cells producing BE1 and BE2 (Figure 1). During three to nine hours post induction, the growth of BE3 gradually increased; however, that of BE1 and BE2 remained noticeably stunted until approximately 12 h after induction, when a rapid population increase was observed. The overnight biomass production of BE1 and BE2 was about 17% higher than BE3 but roughly 26% lower than that of the strains carrying full-length BmpA (Figure 1). The observation was extended to 30 h, but no significant change was recorded.

### 2.2. Localization of Fusion Proteins

**Figure 2:**
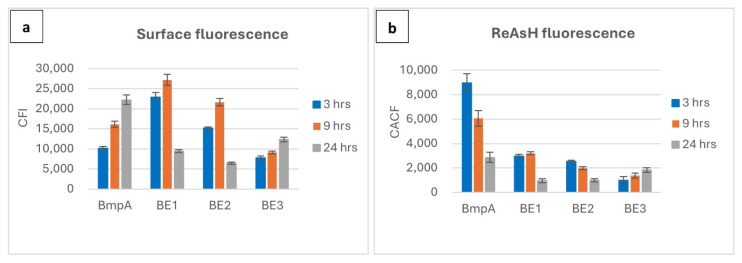
Quantitative comparison on surface and intracellular fluorescence expression of *L. lactis* carrying homologous and fusion proteins over three time points (3, 9, 24 h post induction). Color of columns: blue—3 h, orange—9 h, gray—24 h. (**a**) Surface fluorescence, y-axis: corrected fluorescence intensity of Ni-NTA-Atto 488 (His-tag); (**b**) intracellular fluorescence, y-axis: corrected average cell fluorescence of ReAsH (Tetra-cysteine tag).

Detection of full-length BmpA by immunodetection in the cellular extract, but not in the concentrated cultivation medium, was previously reported and confirmed by a fluorescence shift towards higher levels in flow cytometry assays and by the observance of red fluorescence under the microscope [19]. For BmpA cells, surface fluorescence increased over time, while the intracellular fluorescence was highest at three hours and significantly reduced at later time points. There were no differences in the fluorescence level of stained and unstained negative controls (uninduced cells) at any time point. Among the three fusion constructs, only BE3 showed a temporal pattern similar to BmpA for secretory/displayed protein (Ni-NTA-Atto 488-HisTag); however, the strength of the signals was considerably lower. Three and nine hours post-induction, the average ReAsH-TC fluorescence of BE3 was 9 and 4.4 times weaker than those emitted by BmpA, respectively. The other two strains, BE1 and BE2, displayed similar kinetics with a relatively high surface fluorescence intensity at three hours and a peak at nine hours, before declining steeply at twenty-four hours (Figure 2a).

We observed different patterns concerning intracellular signals of BE1, BE2, and BE3 cells at different time points. At three hours post induction, red fluorescence signals were plentiful and ubiquitous in the cytoplasm of BE1 and BE2 cells; however, they were not visually detected in samples cultivated for up to 24 h. For BE3 cells, fluorescence signals were feeble at three hours but became more pronounced at 24 h (Figure 2b). Histograms and fluorescence microscopy data are shown in Supplementary Figure S1.

### 2.3. Modification of Mature Domain Targeting Signals

**Figure 3:**
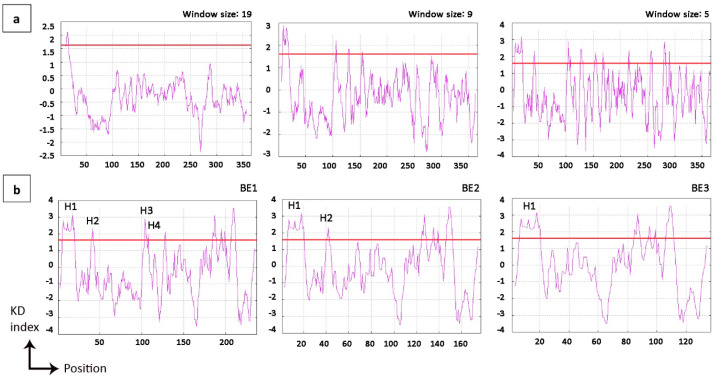
Hydropathy plot of BmpA and fusion proteins. (**a**) BmpA conjugated C-terminally with hexa-histidine and tetra-cysteine tags, 366 amino acids in length. (**b**) BE1, BE2, and BE3 (126, 66, and 26 amino acids at the N-terminus of BmpA fused with HPV16 E7 and C-terminally tagged with hexa-histidine and tetra-cysteine), window size = 5. Red line: threshold +1.6. Graph was created by ProtScale Expasy.

Based on the observation that the length of the homologous fusion partner (26, 66 and 126 amino acids) influences secretion and display efficiencies, we attempted to identify and functionally verify mature domain targeting signals (MTS; [11]) in these fusion constructs. Hydropathy plots of BmpA were displayed at three different spanning sizes of 19, 9, and 5 residues. For all window sizes, the signal peptide of BmpA (residues 1–26) appeared with hydrophobic peaks greater than the +1.6 threshold (Figure 3a). At window span 5, the hydrophobic profile showed that protein BE1 contains four hydrophobic patches, H1–4, with H1 located within the signal peptide and H3 and H4 only separated by two amino acids, Q111 and D112. The fusion protein BE2 contains the signal peptide of BmpA and the following 40 amino acids, encompassing the H1 and H2 patches. The shortest fusion protein BE3 contains only the signal peptide of BmpA and thus only hydrophobic patch H1 (Figure 3b).

**Figure 4:**
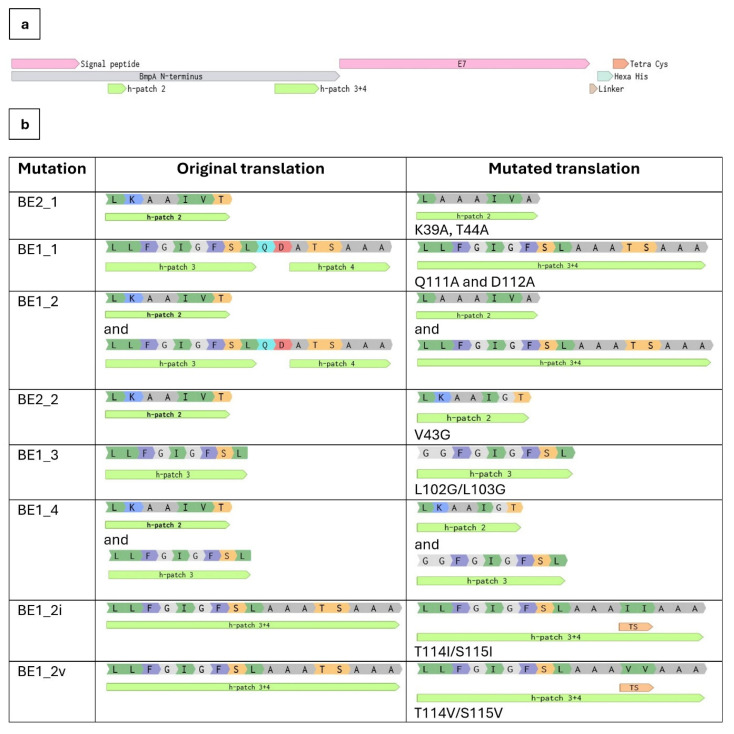
Site-directed designation for mutations. (**a**) Sequence map of BE1 fusion construct. Hydrophobic patches (signal peptide—H1, H2, H3, H4) were identified by Kyte–Doolittle hydropathy evaluation and annotated accordingly. (**b**) List of mutations created using site-directed mutagenesis.

Site-directed mutations were introduced in the truncated N-terminal domain of two fusion constructs BE1 and BE2 for hydropathy modification based on in silico evaluation. BE1_1 showed increased hydrophobicity at H3-H4 due to the mutations Q111A and D112A; BE1_2 had higher hydrophobicity at both H2 and H3-H4 due to mutations K39A, T44A, Q111A, and D112A. In the variants with putatively reduced hydrophobicity, BE1_3 had a weakened stretch at H3 because of the mutations L102G/L103G; BE1_4 had decreased hydrophobicity at both H2 and H3 due to the additional mutation V43G at H2. In fusion protein BE2, H2 was the only MTS, and the predicted profile of variant BE2_1 contained an increased hydrophobic patch due to mutations K39A and T44A, while BE2_2 exhibited reduced hydrophobicity due to mutation V43G. After the first evaluation (not depicted separately), additional mutations were introduced into the construct BE1_2; BE1_2i and BE1_2v possessed additional modifications at H4 (T115I/S116I and T115V/S116V, respectively), in order to further increase the hydrophobicity of these motifs. Table 1 contains a full list of generated variants, details of each mutation are given in Figure 4, and the effects of the introduced amino acid changes on hydropathy predictions at focused hydrophobic patches are shown in Supplementary Figure S2.

Table 2 contains the calculation of molecular weight, theoretical isoelectric point (pI), instability index, aliphatic index, and grand average of hydropathicity (GRAVY) of all produced proteins, including the homologous protein BmpA, the heterologous protein E7, the fusion proteins BE1, BE2, and BE3, and all variants based on these. Almost all variants of the BE1 backbone (BE1_1, BE1_2i, BE1_2v, BE1_3, and BE1_4) share a theoretical pI of 6.43; two variants of the BE2 backbone have a pI of 6.03 (Table 2). The instability index estimates protein stability in vitro, according to which proteins with an instability index lower than 40 are likely to be stable in test tubes [20]. Proteins based on the same backbones tended to share consistent stability. All variants modified from the BE1 backbone were predicted to be stable (index 31.90–34.98). BE2 backbone variants displayed a higher instability index (41.82–42.30), and the shortest fusion protein, BE3, showed the highest index among all variants (45.99). All proteins had high relative occupancy of aliphatic side chains (all above 70), which implied high thermostability and high hydrophobicity [21].

The growth patterns of the cells producing modified fusion proteins shared high similarity with their parental strains BE1 and BE2 (Supplementary Figure S3). A notable delay in growth was observed during the early stages after nisin was added, which continued for approximately 9 h before cell growth resumed. Strains producing the full-length wildtype protein BmpA and the fusion construct BE3 showed an incline over time, with higher values for BmpA than BE3 for surface display at all time points. All cell lines originating from BE1 showed higher levels of protein display at three and nine hours than the highest level of the homologous-protein producer, BmpA cells.

**Figure 5:**
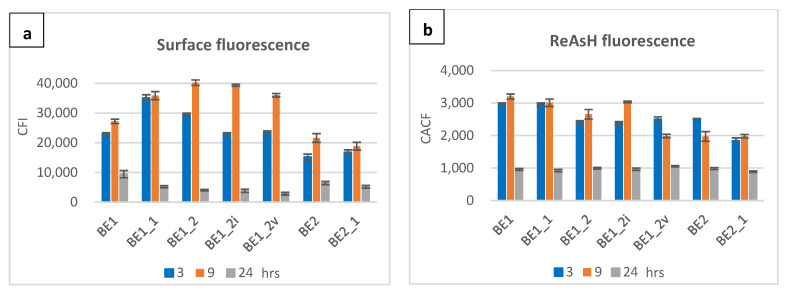
Quantitative comparison on surface and intracellular fluorescence protein expression of alanine variants at 3, 9, and 24 h are included. (**a**) Quantitative comparison of surface expression and (**b**) quantitative comparison of intracellular expression: blue bar—three hours; orange bar—nine hours; light gray bar—24 h.

Figure 5a highlights surface display quantitatively for a better comparison between the strains. BE1 variants showed a proficient surface display at three and nine hours after induction. Two variants, BE1_2 and BE1_2i, showed more than 48% higher surface expression compared to their parent BE1. At three hours, BE1_1 showed the highest surface fluorescence, followed by BE1_2. At nine hours, the highest values were shown by BE1_2, followed by its variants, BE1_2i and BE1_2v, and their parental strain, BE1_1. Intracellular ReAsH-TC fluorescence emitted by all strains was compared as well (Figure 5b). A lower intracellular signal was observed in BE1_2v at nine hours, while the surface fluorescence at the same time point was high. High cytoplasmic fluorescence was seen in several strains, including BE1_2i, BE1_1, BE1, and BE1_3. Compared to all its descendent modified strains, the original BE1 showed weaker surface display and abundant intracellular accumulation at both three and nine hours after induction. Data on the fluorescence shifts in flow cytometry and visual red fluorescence under microscopy are in the Supplementary Materials (Supplementary Figures S4 and S5).

## 3. Discussion

The overexpression of proteins, particularly heterologous proteins, has been shown to trigger a stress response in bacteria [24,25], leading to similar behavior as during osmotic or acidic pressures in the stationary phase [26]. The observed growth behavior is consistent with a stress response in the cells overproducing heterologous proteins but not homologous proteins, as the reduced growth performance and delayed transition into exponential growth post-induction was observed in all BE1 and BE2 variants but not for BmpA or the parent strain NZ9000, which does not overproduce any protein except for the selection marker for plasmid stabilization (Figure 1). Cells producing B3 were afflicted in a different manner, showing a steadier but slower growth pattern and a lower biomass yield after 24 h, suggesting even more pronounced complications due to the overproduction of a heterologous protein.

The efficiency of fusion protein production and display was strongly influenced by the length of the homologous fusion partner. BE3 only contained the signal peptide plus five amino acids to include the conserved cysteine for membrane attachment (26 amino acids) of BmpA. Construct BE2 contains an additional 40 amino acids, 66 in total, and BE1 contains an additional 100 homologous residues for a total of 126 amino acids. Cells producing BE1 and BE2 exhibited two- to three-times higher fluorescence signals from surface-exposed proteins than those producing BE3 at three and nine hours post-induction (Figure 2), with a peak at nine hours post-induction. Cells harboring the longer homologous sequence BE1 displayed 1.2–1.5 times higher surface protein levels at these time points, with a greater difference observed at three hours (Figure 2). These observations suggest that N-terminal homologous regions of 40 or more amino acids after the signal peptide facilitate processing of the translated peptide chain by the cellular components towards translocation and attachment to the membrane. The retarded growth in this phase suggests that some level of stress response, such as an induced overproduction of secretion-associated chaperones, may be required for this, and that the re-routing of cellular resources for these processes causes a reduction in growth. The eventual onset of exponential growth after approximately 12 h appears consistent with a relaxation of these efforts after the overproduced fusion proteins are cleared from the cytoplasm. Cells overproducing the BE3 construct exhibited a generally reduced growth, along with both low surface attachment and low intracellular accumulation at three and nine hours, with a small peak at 24 h. The absence of additional homologous regions following the signal peptide may result in sub-optimal interaction of the heterologous (viral) protein with cellular components (chaperones) responsible for the maintenance of fusion proteins in a translocation-competent unfolded state and for transport to the translocons. This could conceivably lead to an even higher level of unfolded-protein response and a subsequent clearing of accumulated misfolded proteins from the cytoplasm by—presumably—degradation.

Chatzi et al. revealed a correlation between translocation efficiency and the presence of hydrophobic patches, named MTSs, in *E. coli* [11]. These patches are thought to provide interfaces for interactions with cellular components such as secretion-associated chaperones. We have identified such hydrophobic patches (H1 located within the signal peptide, H2, H3, and H4) in the amino acid sequence of BmpA and looked to verify and improve their functionality by exchanging individual amino acids to adjust local hydrophobicity to higher or lower scores. Selected amino acids were substituted against alanine or glycine to minimize structural interference, since these two amino acids represent the simplest side-chain elements. Glycine is the smallest proteogenic amino acid, while alanine is an aliphatic amino acid with one methyl group as side chain, which is generally considered neutral to the functionality of proteins [27]. Even though the distance between alanine and glycine on hydrophobicity scales is not extreme, alanine is considered to be hydrophobic, while glycine is considered to be hydrophilic. The replacement of several hydrophilic residues in the original constructs by alanine was aimed to enhance the hydrophobicity of the corresponding MTSs, and the replacement by glycine was expected to render the potential MTSs less hydrophobic and presumably less functional. The growth patterns of cells harboring modified fusion proteins closely resembled those of their parental strains, BE1 and BE2 (Supplemental Figure S1). BE1 and all its descendant variants (BE1_1, BE1_2, BE1_2i, BE1_2v, BE1_3, BE1_4) exhibited comparable surface attachment of targeted proteins, which was generally higher than those of BE2 and its descendants (BE2_1, BE2_2) (Figure 5a), suggesting that the quantity of hydrophobic patches is the most influential factor for export and attachment. The hydropathy profile of the patches also had some impact, but to a smaller degree. Modifications to alanine (increasing hydrophobicity of the MTSs) did improve translocation, whereas modifications to glycine did not result in a poorer performance compared to their reference strain; the surface fluorescence levels of BE1_3, BE1_4, and BE2_1 were almost the same as in their parent strains. Variants BE1_2i and BE1_2v, with the most significant hydrophobicity in H3 and H4, performed best in protein translocation onto the cell membrane. Between these two variants, BE1_2v showed less accumulation inside the cells, suggesting a complete clearing from the cytoplasm by the translocation machinery (Figure 5b). Further investigations involving more extensive sequence changes in MTSs towards increased hydrophobicity should contribute to a deeper understanding of the involved processes and allow for the establishment of an optimal distribution, size, and hydrophobicity of these signals. The presented findings suggest that a translational fusion between the heterologous protein of interest and a well-secreted native protein harboring at least two or three pronounced MTSs after the signal peptide may be a highly promising approach for improved secretion and cell display. If the size of the fusion construct and consequently increased metabolic load should become a concern, the engineering of designed MTSs into the sequence of a heterologous protein of interest can be an alternative, provided that the introduced sequence changes do not interfere with the natural functionality of the protein in question.

## 4. Materials and Methods

### 4.1. Chemicals and Media

All chemicals and media were of the highest available grade of purity and were obtained from Sigma-Aldrich (St. Louis, MO, USA), except where indicated.

### 4.2. Strain Constructions

**Figure 6:**
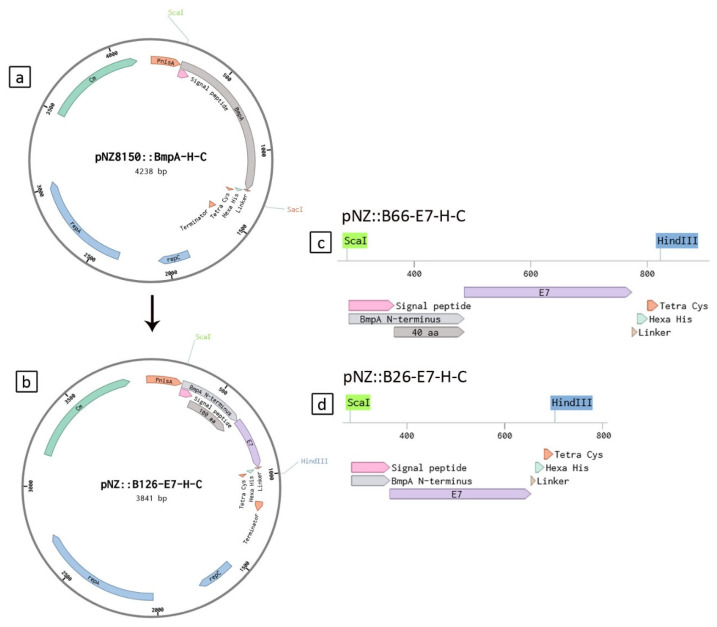
Maps of plasmid constructs. (**a**) pNZ8150 with the gene for the membrane-anchored protein BmpA. (**b**) pNZ8150 with the sequence encoding the first 126 N-terminal amino acids of BmpA fused with the gene for the heterologous oncoprotein E7 from HPV-16. (**c**, **d**) Truncated constructs of the insert from the plasmid in b, containing the first 66 N-terminal amino acids of BmpA, or only the first 26 amino acids (the signal peptide) of BmpA, and E7.

The plasmid vector pNZ8150:BmpA (containing the coding sequence for the basic membrane protein A with a polyhistidine and a tetracysteine tag) was described previously [19]. The sequences encoding three truncated N-terminal domains of BmpA (B126, B66, B26) were amplified from genomic DNA of *L. lactis* NZ9000 using PCR with suitable primer pairs (see Supplementary Table S1). The HPV16 E7 oncoprotein coding sequence extended with a GSG linker, hexa-histidine tag, and tetra-cysteine tag at the C-terminus was synthesized by BioCat (Heidelberg, Germany). Overlapping recognition sites for the endonuclease *Bsa*I were incorporated into both open ends of all sequences encoding BmpA fragments and the E7 oncoprotein to enable Golden Gate assembly with the pNZ8150 plasmid (MoBiTec, Göttingen, Germany). Sequences encoding each truncated BmpA fragment were separately mixed with the synthesized E7 gene and pNZ8150 plasmid DNA in one-pot reactions following the 24-Fragment Golden Gate Assembly protocol from New England Biolabs (Ipswich, MA, USA). The multi-fragment ligations yielded in three hybrid fusion vectors, namely pNZ8150:BE1, pNZ8150:BE2, and pNZ8150:BE3, encoding the BmpA fragments of 126, 66, and 26 amino acids, respectively (Figure 6).

Site-directed mutagenesis was used to introduce point mutations into the fusion constructs. Plasmid pNZ8150:BE1 was used as the template for six variants, and plasmid pNZ8150:BE2 was used for two variants. For each variant, two primers facing in opposite directions and containing an overlap were designed, with a mutation site containing 2–6 modified nucleotides on the forward primer (Supplementary Table S1). PCR reactions were performed using Phusion^®^ High-Fidelity PCR Master Mix with HF buffer (New England Biolabs) to generate linearized DNA fragments carrying the mutations. PCR products were separated by gel electrophoresis (TAE buffer, 0.8% agar *m*/*v*), extracted, and purified using the Monarch^®^ DNA Gel Extraction Kit (New England Biolabs). A total of 150–200 ng of purified linear PCR product was subjected to *Dpn*I endonuclease digestion with Tango buffer (ThermoFisher Scientific, Waltham, MA, USA) at 37 °C for one hour, followed by inactivation at 80 °C for 20 min. Golden Gate Assembly reactions or *Dpn*I-digested PCR products generated with the mutagenesis primers were transformed into electroporation-competent *E. coli* JM101 using a MicroPulser Electroporator (Bio-Rad, Hercules, CA, USA). The cells were mixed with 950 μL recovery media (0.5% (*w*/*v*) yeast extract, 2% (*w*/*v*) tryptone, 10 mM NaCl, 2.5 mM KCl, 20 mM MgSO_4_, 0.5% D-Glucose (*w*/*v*), pH 7.5), rested on ice, incubated at 37 °C for 1–2 h before being plated on LB media solid plates containing 1.5% m/v agar and 10 μg/mL chloramphenicol, and incubated at 37 °C overnight. Five to ten colonies were selected for colony PCR of the respective inserts. Two strains displaying correct PCR bands were inoculated in liquid LB media for plasmid isolation using the Monarch^®^ Plasmid Miniprep Kit (New England Biolabs) and sent for Sanger sequencing (Microsynth, Balgach, Switzerland). Pure plasmids with verified sequences were transformed into electro-competent *L. lactis* NZ9000, generated following the protocol [28]. A full list of the combinations of *L. lactis* strains and plasmids is available in Supplementary Table S2.

### 4.3. Cultivation and Protein Production

*E. coli* strains as intermediate vector recipients were grown in LB medium in glass eprouvettes at 37 °C and 200 rpm. Cultivation of *L. lactis* and induction for protein production was based on the supplier’s manual for the NICE^®^ pNZ8150 expression system and followed the previously established conditions [19,29]. *L. lactis* NZ9000 cells (MoBiTec GmbH) were grown in M17 medium with 0.5% glucose (GM17) at 30 °C without agitation. Except for wildtype strains, all media were supplemented with 10 μg/mL chloramphenicol. *L. lactis* cells were recovered from glycerol stock (1:100 inoculation) and grown overnight. For induction, the cells were diluted in a fresh medium at a ratio of 1:20 and grown until the optical density at 600 nm wavelength (OD_600_) reached 0.5–0.6 (Lambda 35 UV/Vis Spectrometer, PerkinElmer, Waltham, MA, USA), before 25 ng/mL nisin A from *L. lactis* (Sigma-Aldrich) was added. Cells were harvested at 3, 9, and 24 h after induction and stored at 4 °C for further experiments. Cell growth was evaluated based on OD_600_ values (mean of triplicates) at each time point. Negative controls, including uninduced samples and uninduced/induced wildtype strain, were included.

### 4.4. Hydropathy Evaluation

The prediction of the hydropathic character of proteins was based on the Kyte–Doolittle hydrophobicity scale [30] using ProtScale (Expasy, Swiss Institute of Bioinformatics, Lausanne, Switzerland), which evaluates hydrophilic and hydrophobic properties of a protein based on the hydropathy index of its compositions and presents them in the form of a plot displaying the average hydropathic evaluation along the amino acid sequence. For each amino acid position, the algorithm obtains a numerical mean of the hydropathy index of the surrounding amino acids within the customized window size. A midpoint line, which represents the middle value of the hydrophobic portion (upper part) and hydrophilic portion (lower part), is delineated. The choice of segment length (span/window setting) determines each residue’s value by averaging its surrounding residues within the window size [31]. Adjusting the window size based on protein physiology enhances prediction precision, with smaller sizes like 5–7 revealing hydrophilic regions and larger sizes like 19–21 highlighting transmembrane domains. For this study, a window size of 5 was selected after examining plots from 3 to 21, aiming to distinguish hydrophobic and hydrophilic segments and identify hydrophobic patches using 1.6 as transmembrane threshold.

### 4.5. Staining and Detection of Membrane-Anchored and Intracellular Proteins

The detection of membrane anchored proteins was based on the high-affinity interaction between poly-histidine tags with NTA-Atto 488 conjugated with nickel (Ni-NTA-Atto 488, Merck KGaA, Darmstadt, Germany) and performed as published previously [19]. In brief, each sample of 4 × 10^9^ cells were washed twice with 500 µL ice-cold PBS and stained with 2.5 μM Ni-NTA-Atto 488 in PBS for 1.5 h on ice. The cell pellet was then washed three times with ice-cold PBS, finally dissolved in 100 μL ice-cold PBS, and kept on ice until screening. Unstained samples and stained cells containing an empty vector were processed analogously as negative controls. Cells were loaded into a CytoFLEX S flow cytometer (Beckman Coulter, Brea, CA, USA), and protein display was evaluated based on fluorescence emission (band-pass filter 488–525 nm).

The process for staining, detection, and visualization of intracellular proteins in *L. lactis* was described previously [19]. This process consists of (1) the blocking of surface-anchored tetra-cysteine tags using 5,5′-dithio-bis-(2-nitrobenzoic acid) (Ellman’s reagent, DTNB) and (2) the staining of tetra-cysteine-tagged proteins with the membrane-permeable biarsenical compound Resorufin Arsenic Hairpin-binder (ReAsH-EDT_2_; Cayman, Ann Arbor, MI, USA). Each sample of 4 × 10^9^ cells was washed twice with PBS and incubated with 1 mL DTNB solution (4 mM DTNB, 50 mM sodium acetate) at 37 °C for 45 min. The cells were washed twice with PBS before being treated with 500 mL 2,3-dimercaptopropanol (BAL) 650 μM in PBS for 30 min at 37 °C before being washed twice with PBS and stained with 2.5 μM ReAsH-EDT_2_ in PBS for 30 min at 37 °C. After staining, the cell pellet was washed two times with 500 μL BAL 250 μM in PBS before finally being dissolved in 100 μL PBS and stored at 4 °C until further screening. Unstained samples, stained empty-vector cells, and stained wildtype cells were processed analogously as negative controls. The samples were screened in a live-cell epi-fluorescence microscopy system (Leica DMI6000B), lamp EL6000, objective HCX PL APO 63×/1.40 oil with Leica N2.1 filter cube (BP 515–560; Leica Microsystems, Wetzlar, Germany).

### 4.6. Data Analysis

Data analysis was performed as described in [19]. In short, surface-localized proteins were quantitatively evaluated given fluorescence intensity outputs from CytExpert 2.4, and calibrated median fluorescence intensity (CFI) was used for evaluation and graphic presentation. Histogram displays were exported using Floreada (https://floreada.io/, accessed on 30 September 2022). Visual presentations and calculation of intracellular protein quantity were performed using LAS X 3.3.0.16799 and Image J 1.53q. Calibrated average cell fluorescence (CACF) was used for evaluation and graphic displays.

## Figures and Tables

**Table 1:** List of *L. lactis* strains overproducing fusion protein constructs and modifications. Background is shaded for legibility.

Strains	Protein of Interest
**BmpA**	Recombinant BmpA with 6-His and 4-Cys tags
**BE1**	Fusion protein: N-terminal 126 aa of BmpA with full-length E7 with 6-His and 4-Cys tags
**BE2**	Fusion protein: N-terminal 66 aa of BmpA with full-length E7 with 6-His and 4-Cys tags
**BE3**	Fusion protein: N-terminal 26 aa of BmpA with full-length E7 with 6-His and 4-Cys tags
**BE1_1**	BE1 fusion protein with modifications of Q111A and D112A in between H3 and H4
**BE1_2**	BE1 fusion protein with combined modifications of K39A and T44A at H2 and Q111A and D112A in between H3 and H4
**BE1_2i**	BE1_2 protein with additional modifications at H4: T114I and T115I
**BE1_2v**	BE1_2 protein with additional modifications at H4: T114V and T115V
**BE1_3**	BE1 fusion protein with modifications of L102G and L103G at H3
**BE1_4**	BE1 fusion protein with combined modifications of V43G at H2 and L102G and L103G at H3
**BE2_1**	BE2 fusion protein with modifications of K39A and T44A at H2
**BE2_2**	BE2 fusion protein with modifications of V43G at H2

**Table 2:** Computation of several physico-chemical parameters of proteins by ProtParam. Background is shaded for legibility.

Protein	AA	Mol. Weight (Dalton)	Theor. pI (*)	Instability Index (**)	Aliphatic Index	GRAVY (***)
**B**	366	38,453	8.75	12.93	74.13	−0.312
**E7**	111	12,219	5.31	47.53	83.42	−0.132
**BE1**	237	25,595	6.43	34.10	74.56	−0.297
**BE2**	177	19,018	6.03	41.82	85.42	−0.076
**BE3**	137	14,868	6.02	45.99	91.82	0.055
**BE1_1**	237	25,494	6.69	31.91	75.40	−0.252
**BE1_2**	237	25,407	6.43	32.27	76.24	−0.218
**BE1_2i**	237	25,445	6.43	32.27	79.54	−0.173
**BE1_2v**	237	25,417	6.43	32.27	78.69	−0.176
**BE1_3**	237	25,482	6.43	34.62	71.27	−0.332
**BE1_4**	237	25,441	6.43	34.98	70.04	−0.352
**BE2_1**	177	18,931	5.85	42.30	86.55	−0.030
**BE2_2**	177	18,976	6.03	42.30	83.79	−0.102

(*) pI: isoelectric point. Theoretical pI is based on primary sequence. At pI, the net charge of the proteins is zero. (**) Instability index: >40 is classified to be stable in test-tube condition. (***) GRAVY: Grand average of hydropathicity represents hydrophobicity value of a protein. It sums up Kyte–Doolittle’s hydropathy index of each amino acid and divides by the peptide’s length. A positive GRAVY score indicates that the protein is hydrophobic and vice versa [22,23].

## Data Availability

Data and materials generated in this study are available from the corresponding author upon request.

## Supplementary Materials

The following supporting information can be downloaded at https://www.mdpi.com/article/10.3390/ijms26010219/s1.
