# Supplementary material for: Roles of Mature Domain Targeting Signals (MTSs) for Protein Translocation and Secretion in Lactococcus lactis
Source: Int J Mol Sci. 2024 Dec 30;26(1):219. doi: 10.3390/ijms26010219 (PMC11720017; doi:10.3390/ijms26010219)
Supplement: Supplementary file 1 [file ijms-26-00219-s001.zip › ijms-3180979-supplementary.pdf]

Supplementary Material

Supplementary Table 1. List of primers

Name	Sequence	Length	Purpose
Fw_BmpA_8150	GAGGCACTCAGTACTATGAAAAAACGCGTAATCG	34	Amplify BmpA gene, incorporated with labelling tags.
R1_BmpA_8150	GATGGTGACCACTACCTTTTGAAGGAACAG	30	Amplify BmpA gene, incorporated with labelling tags.
Rv2_ BmpA_8150	CAACAATGGTGATGGTGATGGTGACCACTACC	32	Amplify BmpA gene, incorporated with labelling tags.
Rv3_ BmpA_8150	GCTTTTAGCAACAAGGACCGCAACAATGGTGAT	33	Amplify BmpA gene, incorporated with labelling tags.
R4_BmpA_8150	AGCTTGAGCTCGCTTTTAGCAACAAGG	27	Amplify BmpA gene, incorporated with labelling tags.
Fw_B26	GCATAGGTCTCTGTACCATGAAAAAACGCGTAATCGCAG	39	Amplify N-terminal domains of truncated BmpA (B126, B66, B26)
Rv_B26	AATATGGTCTCATCATGTCATGTGAACGACATCCTGC	37	Amplify N-terminal domains of B26
Rv_B66	AATATGGTCTCATCATCCCCCAGCTTTGAAGACCTTC	37	Amplify N-terminal domains of B66
Rv_B126	AATATGGTCTCATCATAAAGTTAGATTTTGGATTATTTTTCGC	43	Amplify N-terminal domains of B126
Fw_U2	GTAAAAACTGACCTTGCAGCAGCAATCGTCGCAGATGCTAATGGTGTAAATGACCG	56	mutation
Rv_U2	AAGGTCAGTTTTTACTTTACCTGAAGTTC	29	mutation
Fw_U34	GGATTCTCTTTAGCAGCAGCGACATCAGCAGCTGCGAAAAATAATC	46	mutation
Rv_U34	TAAAGAGAATCCAATACCAAACAAAAGTTTGTAAC	35	mutation
Fw_D2	CTTAAAGCAGCAATCGGCACCGATGCTAATGGTGTAAATGACCGTTC	47	mutation
Rv_D2	CGATTGCTGCTTTAAGGTCAGTTTTTACTTTAC	33	mutation
Fw_D3	CAACAAGGTTACAAAGGTGGTTTTGGTATTGGATTCTCTTTACAAGATGCG	51	mutation
Rv_D3	TTTGTAACCTTGTTGCTCCGCAGAGTTATAG	31	mutation
Fw_II	CTCTTTAGCAGCAGCGATCATTGCAGCTGCGAAAAATAATCCAAAATC	48	mutation
Fw_VV	CTCTTTAGCAGCAGCGGTTGTGGCAGCTGCGAAAAATAATCCAAAATC	48	mutation
Rv_TS	CGCTGCTGCTAAAGAGAATCCAATACC	27	mutation
Fw_BE7_seq	ACAAGAATTAGTATTGCCTC	20	sequencing
Rv_BE7_seq	CACAGAATGCACACAAAGG	19	sequencing

Fw_8150_MSC	CTTAATTCTATCTTGAGAAAGTATTGG	27	sequencing
Rv_8150_MSC	GTAATTGCTTTATCAACTGCTGC	23	sequencing
Fw_BmpA_seq	CAGGTGTCTTCAGTGAAGC	19	sequencing
Rv_BmpA_seq	GGTCTTTAATGACAGAGTC	19	sequencing

Supplementary Table 2. List of *L. lactis* strains containing plasmids

Strains	Original Strains	Plasmids	Protein of interest
NpB	NZ9000	pNZ8150:BmpA	Recombinant BmpA with 6-His & 4-Cys tags
BE1	NZ9000	pNZ8150:BE1	*Fusion protein*: N-terminal 126 aa of BmpA with full-length E7 with 6-His & 4-Cys tags
BE2	NZ9000	pNZ8150:BE2	*Fusion protein*: N-terminal 66 aa of BmpA with full-length E7 with 6-His & 4-Cys tags
BE3	NZ9000	pNZ8150:BE3	*Fusion protein*: N-terminal 26 aa of BmpA with full-length E7 with 6-His & 4-Cys tags
BE1_1	NZ9000	pNZ8150:BE1B	BE1 fusion protein with modifications of Q111A and D112A in between H3 and H4
BE1_2	NZ9000	pNZ8150:BE1AB	BE1 fusion protein with combined modifications of K39A and T44A at H2 and Q111A and D112A in between H3 and H4
BE1_2i	NZ9000	pNZ8150:BE1ABi	BE1AB protein with more modifications at H4: T114I and T115I
BE1_2v	NZ9000	pNZ8150:BE1ABv	BE1AB protein with more modifications at H4: T114V and T115V
BE1_3	NZ9000	pNZ8150: BE1D	BE1D fusion protein with modifications of L102G and L103G at H3
BE1_4	NZ9000	pNZ8150:BE1CD	BE1CD fusion protein with combined modifications of V43G at H2 and L102G and L103G at H3
BE2_1	NZ9000	pNZ8150:BE2A	BE2 fusion protein with modifications of K39A and T44A at H2
BE2_2	NZ9000	pNZ8150:BE2C	BE2 fusion protein with modifications of V43G at H2

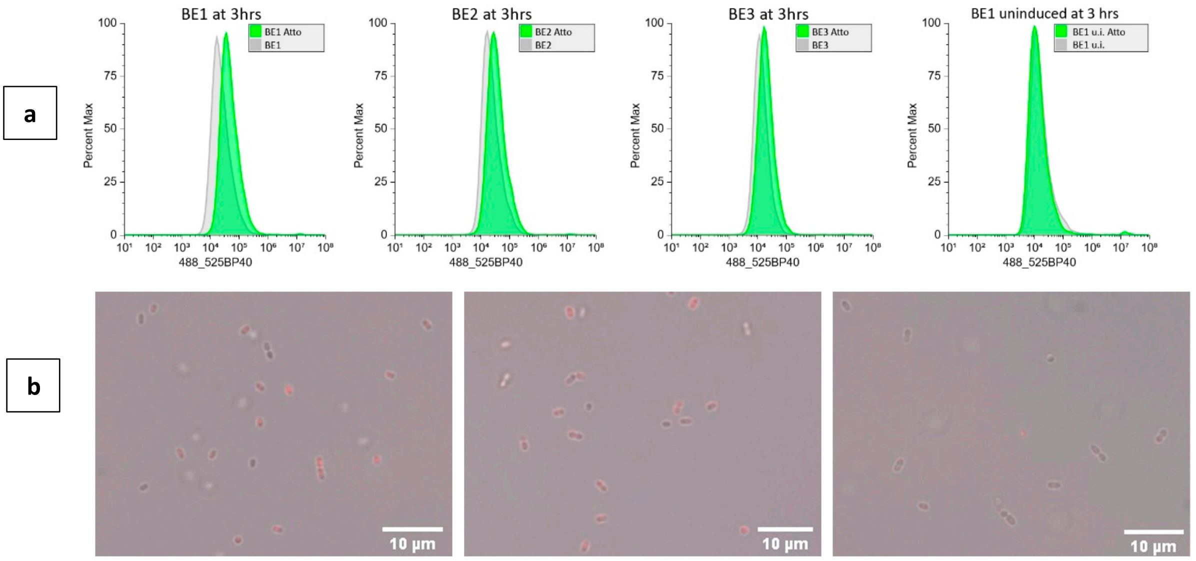

Supplementary Figure S1. Overexpression of hybrid fusion proteins at 3-hour culture. (a) Overexpression of hybrid fusion proteins on the surface, displayed by the overlay histogram of unstained population (grey) and stained population (green) in flow cytometry. (b) Overexpression of intracellular hybrid fusion proteins, displayed by overlay as scaled viewer image of two microscopic channels (brightfield and red fluorescence). The order from left to right is BE1, BE2, BE3.

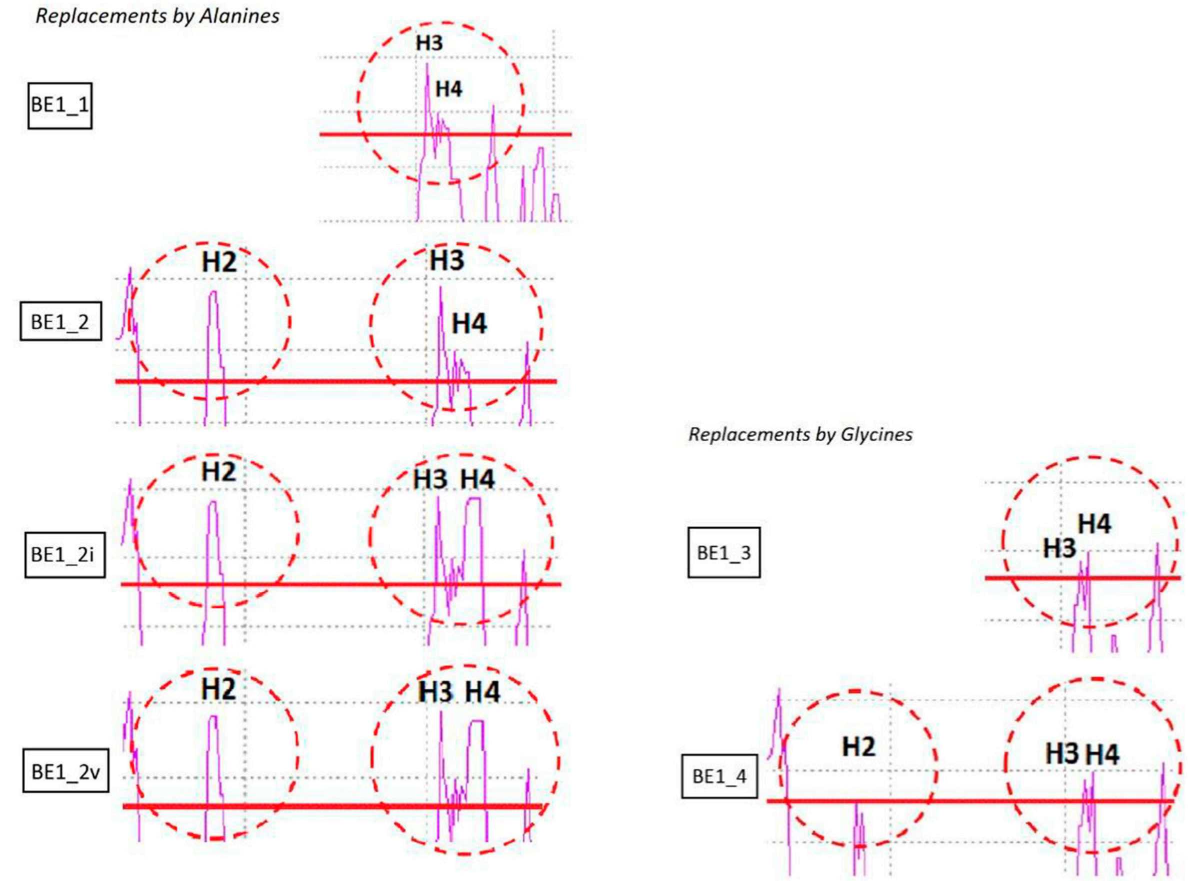

Supplementary Figure S2. Hydropathy plot of BE1-based variants. Replacements by alanine target the increase of hydrophobicity (BE1_1, BE1_2, BE1_2i, BE1_2v). Replacements by glycine target the decrease of hydrophobicity (BE1_3, BE1_4).

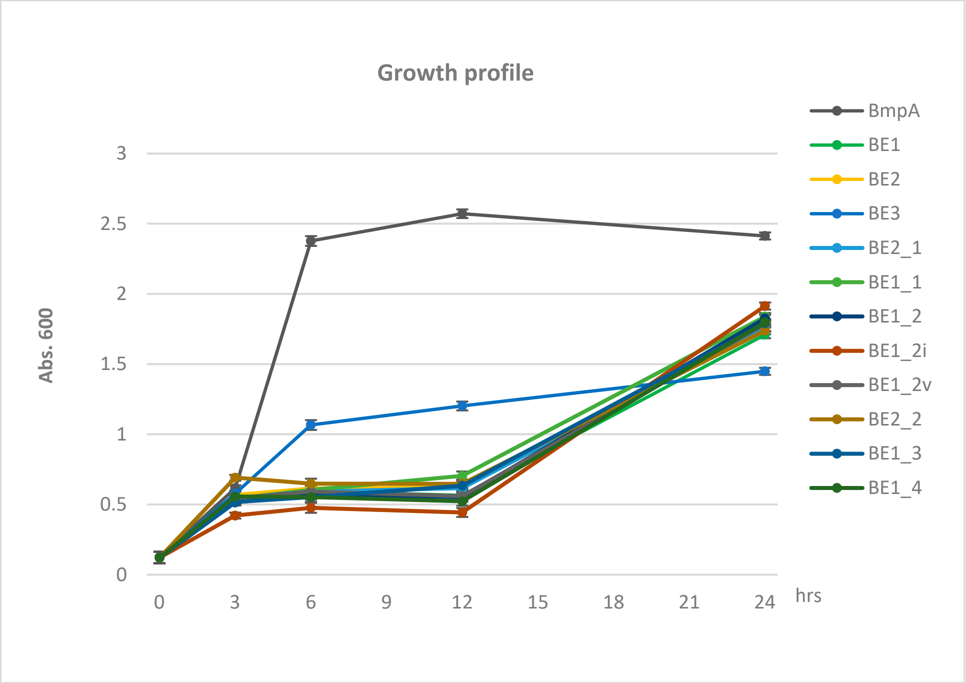

Supplementary Figure S3. Growth profiles of all strains

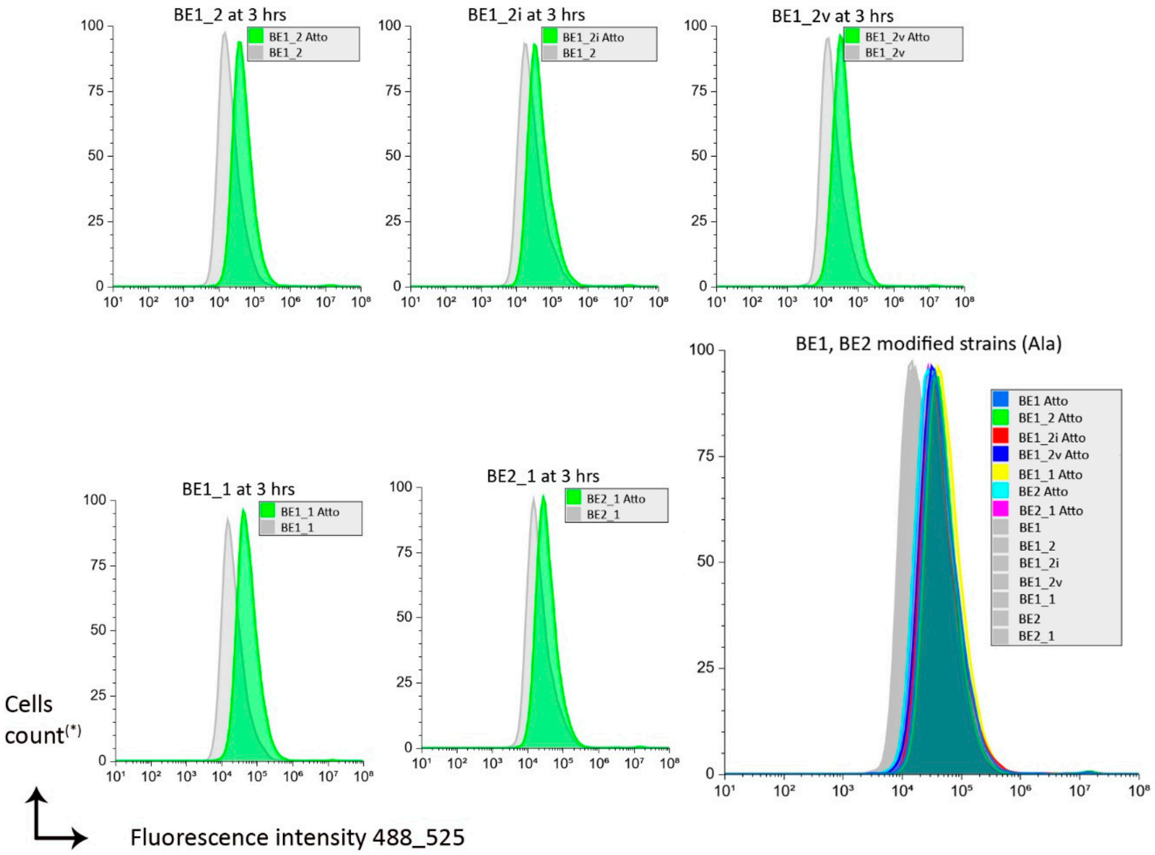

Supplementary Figure S4. Surface expression of cells carrying modified fusion proteins (alanine). Each individual histogram displays the peaks of unstained sample (grey peak) and stained sample (green peak). In the overlay histogram, all unstained and stained samples were combined, in which all unstained samples were displayed by grey colour and stained samples were displayed by different colours. (*) Normalized cells count to one hundred percent.

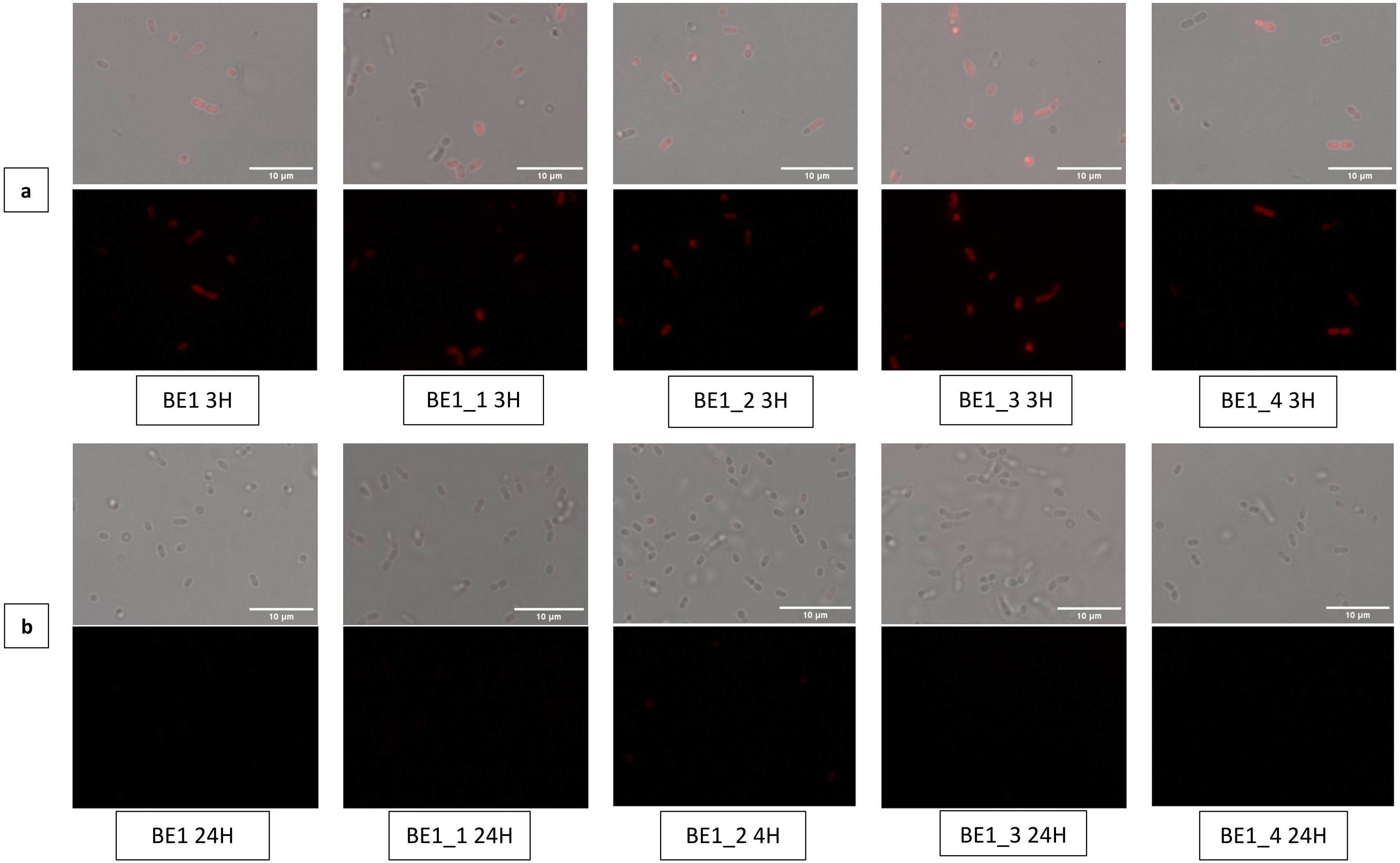
a
10 µm
BE1 3H
BE1_1 3H
BE1_2 3H
BE1_3 3H
BE1_4 3H
b
BE1 24H
BE1_1 24H
BE1_2 4H
BE1_3 24H
BE1_4 24H

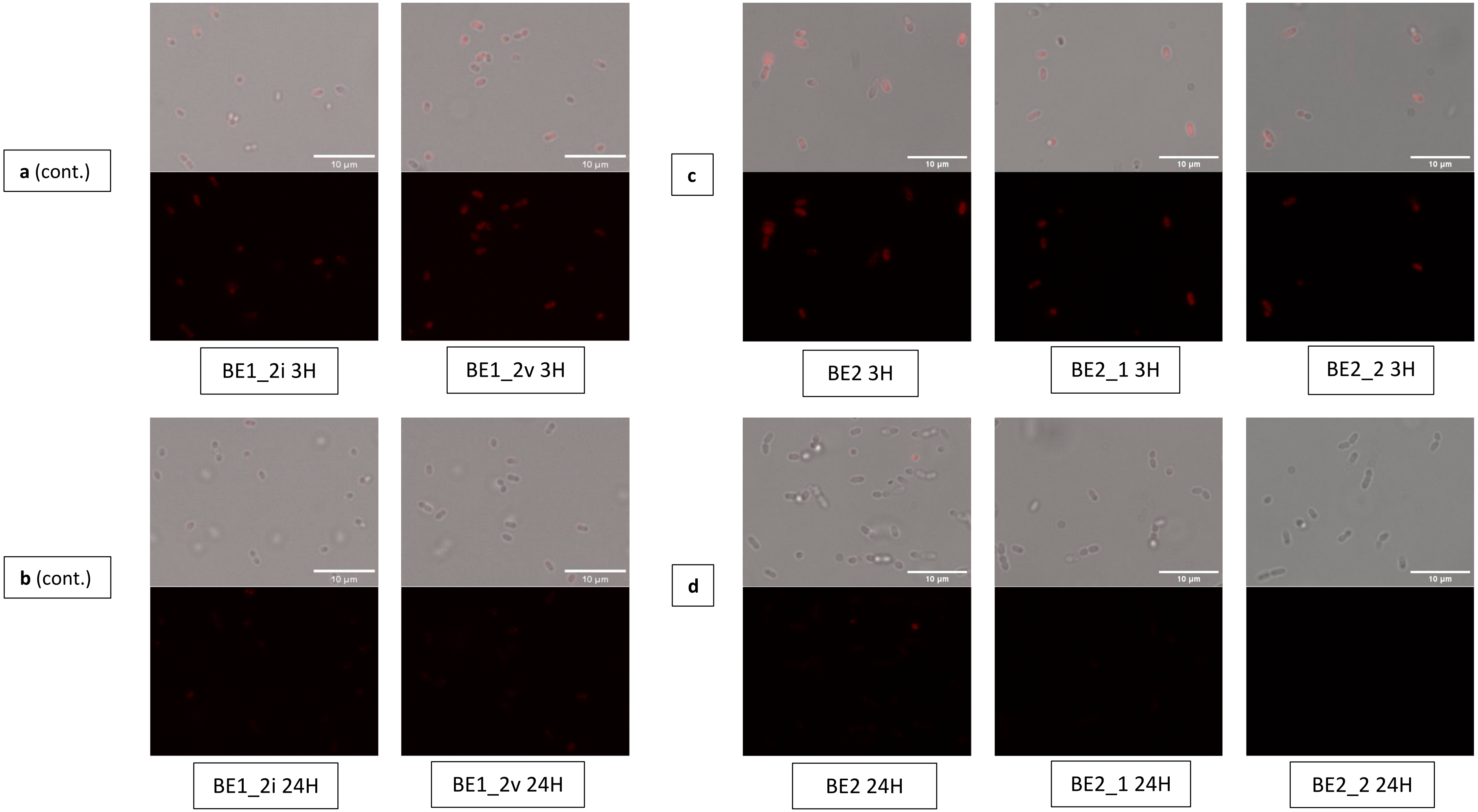

Supplementary Figure S5. Intracellular fluorescence expression of all cell strains producing fusion proteins with modified hydrophobic patches (a) BE1 variations at 3 hours, (b) BE1 variations at 24 hours, (c) BE2 variations at 3 hours, (d) BE2 variations at 24 hours.
